# Kidney morphological parameters measured using noncontrast-enhanced steady-state free precession MRI with spatially selective inversion recovery pulse correlate with eGFR in patients with advanced CKD

**DOI:** 10.1007/s10157-017-1413-x

**Published:** 2017-04-22

**Authors:** Tadashi Otsuka, Yoshikatsu Kaneko, Yuya Sato, Ryohei Kaseda, Ryuji Aoyagi, Suguru Yamamoto, Shin Goto, Ichiei Narita

**Affiliations:** 1 Division of Clinical Nephrology and Rheumatology, Niigata University Graduate School of Medical and Dental Sciences, 1-757 Asahimachi-dori, Niigata, 951-8510 Japan; 2 Tachikawa General Hospital, 561-1 Azayachi, Jojomachi, Nagaoka, Niigata 940-8621 Japan

**Keywords:** SSFP MRI, Cortical thickness, Cortical area, Medullary area

## Abstract

**Background:**

It is well known that atrophic renal changes are associated with chronic kidney disease (CKD) progression, but conventional diagnostic imaging methods such as noncontrast-enhanced computed tomography and magnetic resonance imaging (MRI) have been insufficient for precisely assessing kidney function because they cannot clearly distinguish between the medulla and cortex. Hence, here we used noncontrast-enhanced steady-state free precession (SSFP) MRI with a spatially selective inversion recovery (IR) pulse to improve visibility for renal corticomedullary differentiation and evaluated the association between morphological parameters and kidney function in patients with CKD.

**Methods:**

Kidney corticomedullary contrast ratio, cortical and medullary areas, and minimal cortical thickness of 107 patients with CKD G1–G5 were measured using SSFP MRI with a spatially selective IR pulse and the association between these morphological parameters and kidney function were evaluated.

**Results:**

Corticomedullary contrast ratio was significantly improved on SSFP MRI compared with conventional in-phase T1-weighted gradient-echo MRI and positively correlated with estimated glomerular filtration ratio (eGFR), raw eGFR, and 24-h creatinine clearance. The medullary and cortical areas and minimal cortical thickness also positively correlated with those of kidney functional markers and the age. In patients with CKD and diabetes mellitus (DM), the correlation coefficients between raw eGFR and morphological parameters were higher than those in patients without DM, while minimal cortical thickness was larger in CKD patients with DM with a raw eGFR ≥ 45 mL/min.

**Conclusion:**

Kidney morphological parameters measured with SSFP MRI were clearly correlated with kidney function in patients with CKD, including those with advanced kidney dysfunction.

## Introduction

In chronic kidney disease (CKD), kidney dysfunction progression correlates with reduced bipolar kidney length, kidney volume, and cortical thickness [1]. Kidney biopsy is an effective method for obtaining pathological information and evaluating CKD progression; however, percutaneous kidney biopsy is rather contraindicated for patients with advanced CKD and an atrophic renal cortex. Therefore, non-invasive quantitative and qualitative evaluations such as ultrasonography (US), computed tomography (CT), or magnetic resonance imaging (MRI) have long been attempted to assess morphological changes in the diseased kidney and estimate the etiology, duration, and reversibility of kidney disease.

Among these diagnostic imaging methods, US has often been used in the initial evaluation of CKD patients for its convenience. Measurement of the renal cortical thickness using US was reported to be valuable for estimating residual kidney function in CKD patients and predicting their prognosis [2]. However, its interobserver reproducibility was not guaranteed and its reproducibility for cortical thickness measurements was not satisfactory [3]. Although contrast-enhanced CT is considered an accurate method for measuring renal morphological parameters including renal cortical thickness, its routine use for this purpose is limited due to the potential nephrotoxicity of contrast agents.

Noncontrast MRI was previously considered insufficient to distinguish between the renal cortex and medulla, especially in patients with kidney dysfunction because of an increased T1 relaxation time of the cortex accompanied with decreased kidney function [4, 5], so the evaluation of corticomedullary differentiation required dynamic contrast-enhanced MRI. However, the risk of nephrogenic systemic fibrosis caused by contrast agent use could not be neglected [6]. Recently, noncontrast-enhanced steady-state free precession (SSFP) MRI with a spatially selective inversion recovery (IR) pulse was reported to improve the resolution of renal corticomedullary differentiation in patients without a history of renal disease [7] and in those with CKD [8]. Noda et al. reported a significant association between minimal renal cortical thickness and estimated glomerular filtration rate (eGFR) in patients with CKD using this method. However, the mean eGFR of the participants in the previous report was 79.7 mL/min/1.73 m^2^ (range 36.1–125.9 mL/min/1.73 m^2^), while only 16 of 65 participants had an eGFR < 60.0 mL/min/1.73 m^2^ [8]. The usefulness of this method has not yet been proven in patients with more advanced CKD stages, such as CKD G4 and G5.

Therefore, the purposes of the present study were to assess whether noncontrast-enhanced SSFP MRI with a spatially selective IR pulse improved the visibility of renal corticomedullary differentiation in patients with CKD including those with advanced stages and to investigate the correlation between kidney function and segmental areas of the kidney compartments.

## Materials and methods

### Subjects

In our retrospective study, we identified 107 patients diagnosed with CKD who underwent noncontrast-enhanced SSFP MRI with spatially selective IR pulse at Tachikawa General Hospital between February 2015 and July 2016. Clinical evaluations were conducted within a week after MRI examination. Patients were excluded from the study if they had a single kidney, a large solid/cystic lesion in the kidney, autosomal dominant polycystic kidney disease, acute kidney injury, unacceptable image quality, or insufficient medical information. Patients with a rotated kidney were also excluded because of the possible incorrectness of measuring morphological parameters. The Institutional Review Board of Tachikawa General Hospital approved this retrospective study (approval number 12000056) and the patients provided written informed consent.

### Definition

CKD was defined as an eGFR < 60 mL/min/1.73 m^2^ and/or the presence of proteinuria (urine protein ≥ 0.15 g/gCr or urine albumin ≥ 30 mg/gCr), and these abnormalities of the kidney were present for more than 3 months and were staged according to the 2012 Kidney Disease: Improving Global Outcomes (KDIGO) guidelines [9, 10]. The eGFR values were calculated using the modification of diet in renal disease formula for Japanese patients: eGFR (mL/min/1.73 m^2^) = 194 × [serum creatinine (mg/dL)]^−1.094^ × [age (years)]^−0.287^ × 0.739 (if female) [11]. Body surface area (BSA) was calculated according to the DuBois and DuBois formula [12]. Raw eGFR was calculated using the following equation: raw eGFR (mL/min) = eGFR × BSA/1.73. The diagnosis of diabetes mellitus (DM) was based on American Diabetes Association criteria [13] or on the basis of a history of type 2 DM under dietary intervention or the use of hypoglycemic agents. Patients who received anti-hypertensive agents were defined as those treated for more than 6 months.

### Imaging technique

All MRI examinations for SSFP images were performed with a 1.5-T unit (Vantage Atlas MRT-2003; Toshiba Medical Systems, Tokyo, Japan) using the Atlas SPEEDER™ body coil. The procedure for the MRI imaging was described in the previous study by Kanki et al. [7]. Imaging parameters of the SSFP sequence were as follows: repetition time/echo time (TR/TE) = 4.2/2.1 ms; number of acquisitions = 1; parallel imaging factor = 2; flip angle = 90°; receiver bandwidth = 977 Hz/pixel; slice thickness = 7 mm; field of view = 400 × 400 mm; acquisition matrix = 256 × 256. Noncontrast-enhanced SSFP MRI of the kidney with a spatially selective IR pulse was performed using identical imaging parameters during a single breath hold. A spatially selective IR pulse with a thickness of 130 mm was placed on both kidneys. To measure and compare each parameter of kidney imaging under the same conditions, 1400 ms was defined as a standard TI by reference to a previous report. In addition, in-phase (IP) T1-weighted gradient-echo MR images were obtained to compare the visibility of corticomedullary differentiation as well as the corticomedullary contrast ratio of SSFP MRI with a spatially selective IR pulse. Imaging parameters of the IP sequence were as follows: TR/TE = 240/4.8 ms; number of acquisitions = 1; flip angle = 90°; receiver bandwidth = 488 Hz/pixel; slice thickness = 7 mm; field of view = 400 × 400 mm; acquisition matrix = 320 × 192 [7]. For comparison with conventional IP images at 3.0-T unit, MRI examinations were conducted with GE SIGNA™ Pioneer 3.0 T scanner (GE Healthcare, Piscataway, NJ, USA) using TDI Anterior Array coil and TDI Posterior Array coil.

### Data analyses

**Fig. 1:**
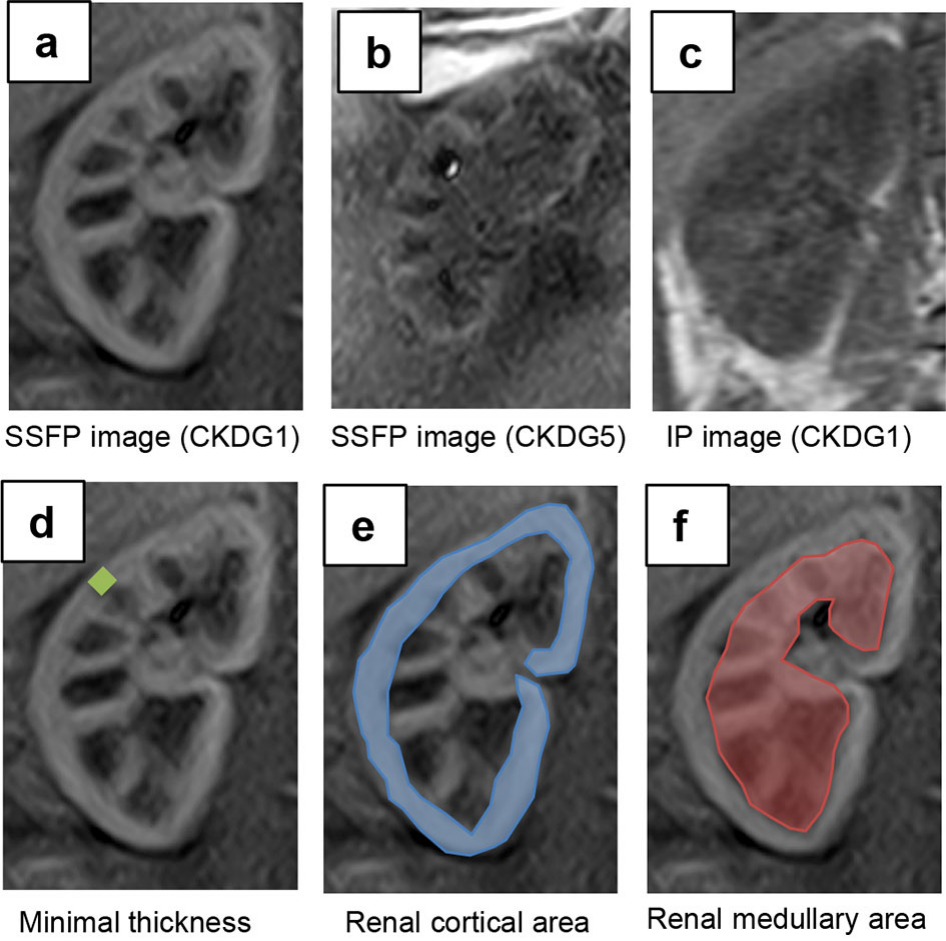
Representative coronal kidney images of noncontrast-enhanced steady-state free precession (SSFP) magnetic resonance imaging (MRI) with spatially selective inversion recovery pulse at 1.5-T unit (**a**, **b**, **d**–**f**) or conventional in-phase MRI at 3.0-T unit (**c**). **a** An SSFP image of a CKD G1 patient. **b** An SSFP image of a CKD G5 patient. **c** A conventional in-phase image of a CKD G1 patient. Minimal cortical thickness, cortical area, and medullary area are shown as a *yellow band* (**d**), *blue* area (**e**), and *red* area (**f**), respectively

All images were reviewed on a clinical picture archiving and communication system workstation monitor (Rapideye Core; Toshiba Medical Systems) by two physicians blinded to the subjects’ clinical information. The physicians selected the larger of the right or left kidney as the measuring object and drew operator-defined regions of interest (ROI) within the cortex and medulla in the target kidney to measure the signal intensity (SI) values of each segment by consensus. ROI measurements were also performed on the IP images. The corticomedullary contrast ratio was calculated from SI values of the renal cortex (SI_cortex_) and renal medulla (SI_medulla_) as (SI_cortex_/SI_medulla_). Additionally, the physicians measured and recorded the minimal cortical thicknesses and coronal sectional areas of the cortex and medulla as shown in Fig. 1.

### Statistical analyses

Corticomedullary contrast ratios between SSFP and IP images were compared using the Wilcoxon signed-rank test. The Pearson product-moment correlation coefficient was used to investigate the correlation between two parameters in each case. Simple associations were performed using unpaired *t* tests or appropriate non-parametric tests for continuous variables and Chi-square tests for categorical variables. The interobserver agreements in relation to morphological parameters were verified by intraclass correlation coefficient (ICC). Value of *P* < 0.05 was considered statistically significant.

## Results

### Characteristics of the study subjects and morphological variables of the kidney

**Fig. 2:**
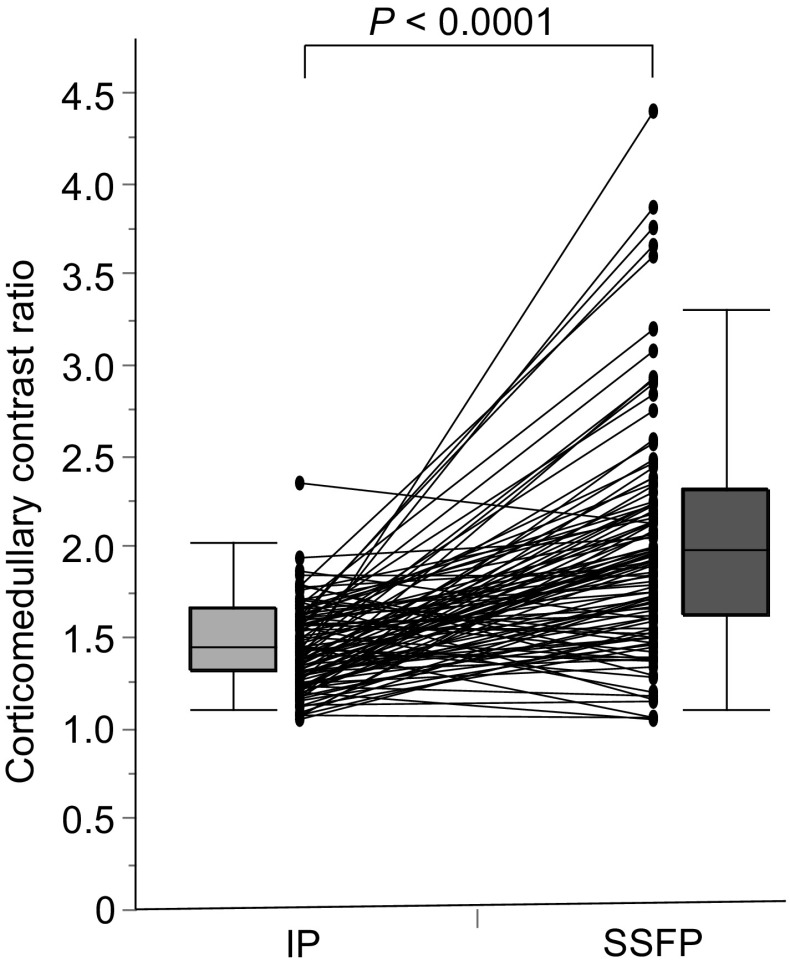
Comparison of corticomedullary contrast ratio. Mean corticomedullary contrast ratio of the patients with chronic kidney disease (*n* = 107) was significantly higher in steady-state free precession (SSFP) images than in in-phase (IP) images

**Fig. 3:**
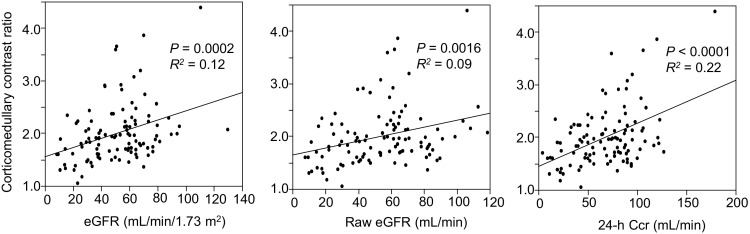
Correlation between corticomedullary contrast ratio and estimated glomerular filtration rate (eGFR), raw eGFR, or 24-h creatinine clearance (Ccr). The corticomedullary contrast ratio was positively correlated with all three markers of kidney function

A total of 107 patients diagnosed with CKD were enrolled in this study. Table 1 presents the background profiles of the entire study population. The median age was 64 years and 60.7% of the patients were men. The mean eGFR, raw eGFR, and 24-h creatinine clearance (Ccr) were 51.1 ± 22.7 mL/min/1.73 m^2^ (range 8.4–130.2), 53.7 ± 25.4 mL/min (range 7.6–118.6), and 67.6 ± 31.7 mL/min (range 4.0–179.0), respectively. The numbers and percentages of patients with each CKD stage were as follows: CKD G1, four (3.7%); G2, 35 (32.7%); G3, 48 (44.9%); G4, 13 (12.1%); G5, seven (6.5%). Anti-hypertensive agents were used in 93 (86.9%) patients, and 73 patients (68.2%) were treated with renin–angiotensin system (RAS) inhibitors, whereas 14 patients (13.1%) were treated with diuretics. Renal corticomedullary differentiation was clearly depicted in SSFP images using standard TI, but not in conventional IP images at even 3.0-T unit MRI (Fig. 1). The mean corticomedullary contrast ratio was higher in SSFP images than in IP images with some exceptional cases (Fig. 2), and the corticomedullary contrast ratio in SSFP images positively correlated with all of the three kidney functional parameters (Fig. 3). Then three morphological variables, minimal cortical thickness, cortical area, and medullary area of all patients were subjected to the validation study of interobserver reproducibility, and each ICC demonstrated a good correlation (Table 2). Average values of the measured kidney morphological parameters are shown in Table 3.

**Table 1:** Clinical characteristics of the patients with CKD (*n* = 107)

Male sex [*n* (%)]	65 (60.7)
Age (years; mean ± SD, range)	64 ± 17, 18–88
Height (m; mean ± SD)	1.61 ± 0.10
Weight (kg; mean ± SD)	62.9 ± 14.9
BMI (mean ± SD)	24.2 ± 4.2
BSA (m^2^; mean ± SD)	1.79 ± 0.25
BUN [mg/dL; median (IQR)]	17.0 (13.6–22.6)
Creatinine [mg/dL; median (IQR)]	1.02 (0.84–1.53)
eGFR (mL/min/1.73 m^2^; mean ± SD, range)	51.1 ± 22.7, 8.4–130.2
Raw eGFR (mL/min; mean ± SD, range)	53.7 ± 25.4, 7.6–118.6
24 h Ccr (mL/min; mean ± SD, range)	67.6 ± 31.7, 4.0–179.0
Urinary protein [mg/day; median (IQR)]	182 (52–865)
CKD stage [*n* (%)]	
G1	4 (3.7)
G2	35 (32.7)
G3	48 (44.9)
G4	13 (12.1)
G5	7 (6.5)
HbA1c (%; mean ± SD)	5.8 ± 0.7
Anti-hypertensive agents [*n* (%)]	93 (86.9)
RAS inhibitor [*n* (%)]	73 (68.2)
Calcium channel blockers [*n* (%)]	49 (45.8)
Diuretics [*n* (%)]	14 (13.1)
Beta-blockers [*n* (%)]	16 (15.0)

*CKD* chronic kidney disease, *BMI* body mass index, *BSA* body surface area, *BUN* blood urea nitrogen, *eGFR* estimated glomerular filtration rate, *Ccr* creatinine clearance, *RAS* renin–angiotensin system

**Table 2:** Interobserver reproducibility in the measurement of kidney morphological parameters

	ICC	Interval for the ICC of 95%	*P* value
Minimal cortical thickness	0.866	0.809–0.906	<0.0001
Cortical area	0.988	0.982–0.992	<0.0001
Medullary area	0.941	0.915–0.956	<0.0001

*ICC* intraclass correlation coefficient

**Table 3:** Measured kidney morphological parameters

Cortical thickness (mm; mean ± SD)	
Maximal	7.5 ± 1.9
Minimal	5.3 ± 1.7
Coronal sectional area (cm^2^; mean ± SD)	
Cortex	12.88 ± 4.21
Medulla	17.24 ± 5.65
Entire kidney	30.11 ± 8.51

### Correlation between kidney morphological parameters and function in patients with CKD

**Fig. 4:**
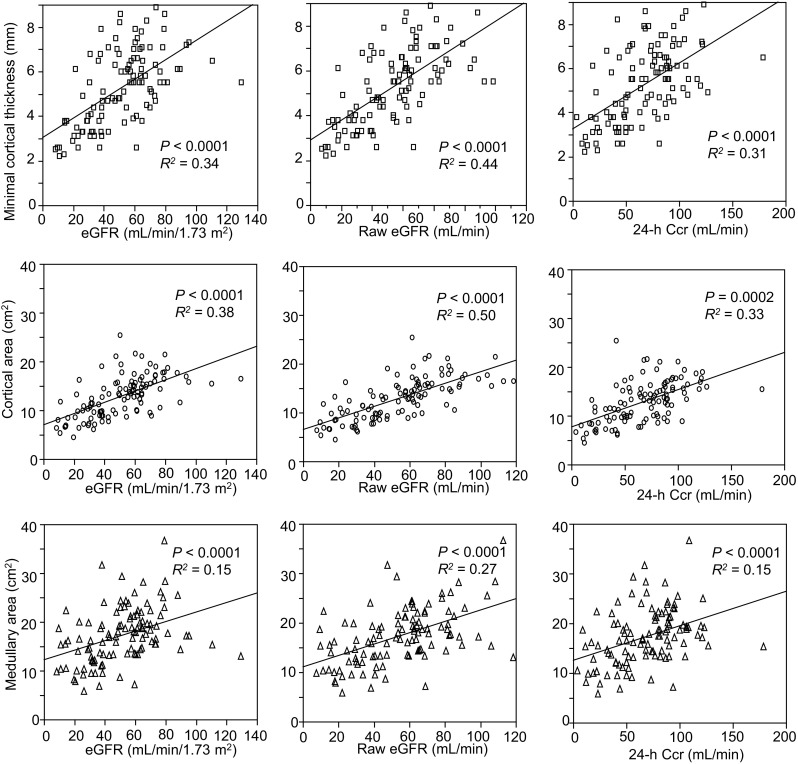
Correlation between kidney function and minimal cortical thickness (*upper*), cortical area (*middle*), or medullary area (*lower*). All three morphological parameters were positively correlated with estimated glomerular filtration rate (eGFR), raw eGFR, or 24-h creatinine clearance (Ccr)

The correlations between kidney morphological parameters and kidney functional markers were assessed by analysis of variance. All of the three parameters were significantly correlated with eGFR, raw eGFR, and 24-h Ccr. Among these kidney morphological parameters, cortical area demonstrated the highest correlation coefficients with all three kidney functional markers. Regarding kidney functional markers, raw eGFR exhibited the highest correlation coefficients with all three kidney morphological parameters (Fig. 4), suggesting that the cortical area is the most efficient parameter for estimating raw eGFR in patients with CKD. Three patients had a unilateral atrophic kidney with volume reduction and a unilateral renal artery stenosis on the same side. In these cases, no significant correlation between minimal cortical thickness of the atrophic kidney and kidney function was observed.

**Fig. 5:**
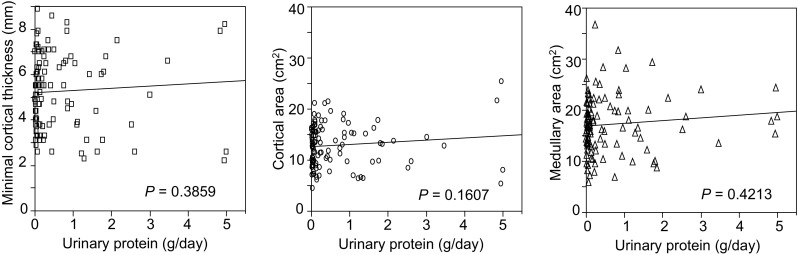
Correlation between 24-h urinary protein amount and minimal cortical thickness, cortical area, or medullary area. There were no significant correlations between 24-h urinary protein amount and kidney morphological parameters

We also analyzed the correlations between daily urinary protein level and each kidney morphological parameter since urinary protein level reflects kidney disease severity. However, no significant correlations were observed among them (Fig. 5).

### Correlation between kidney morphological parameters and age in patients with CKD

**Fig. 6:**
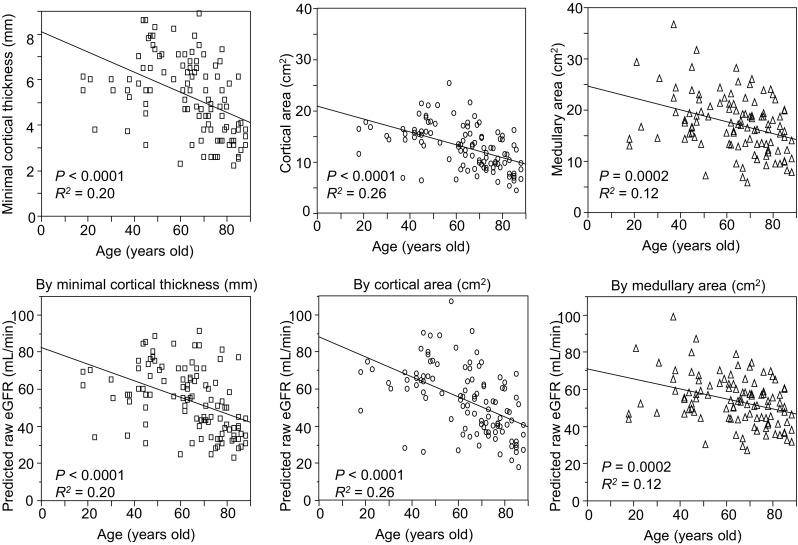
Correlation between age and morphological parameters. All of three morphological parameters, minimal cortical thickness, cortical area, and medullary area (*upper*) were negatively correlated with the age of the CKD patients. Predicted raw eGFR based on each morphological parameter using its peculiar regression line in Fig. 4 also significantly correlated with the age (*lower*). Predicted raw eGFR was calculated as follows: predicted raw eGFR by minimal cortical thickness (mL/min) = 10.06 × minimal cortical thickness (mm) + 0.728 (*left*); predicted raw eGFR by cortical area (mL/min) = 4.3 × cortical area (cm^2^) − 1.363 (*middle*); predicted raw eGFR by medullary area (mL/min) = 2.3 × medullary area (cm^2^) + 13.42 (*right*)

Then we analyzed the correlation between age and each morphological parameter, because GFR is progressively decreased by aging [14, 15] and age-related reduction of eGFR could affect the morphological parameters. Figure 6 shows that negative correlations were observed between age and each morphological parameter. We also estimated original raw eGFR from each morphological parameter using regression line in Fig. 4, and the predicted raw eGFR also significantly correlated with the age.

### Correlation between kidney morphological parameters and function in CKD patients with or without DM

**Fig. 7:**
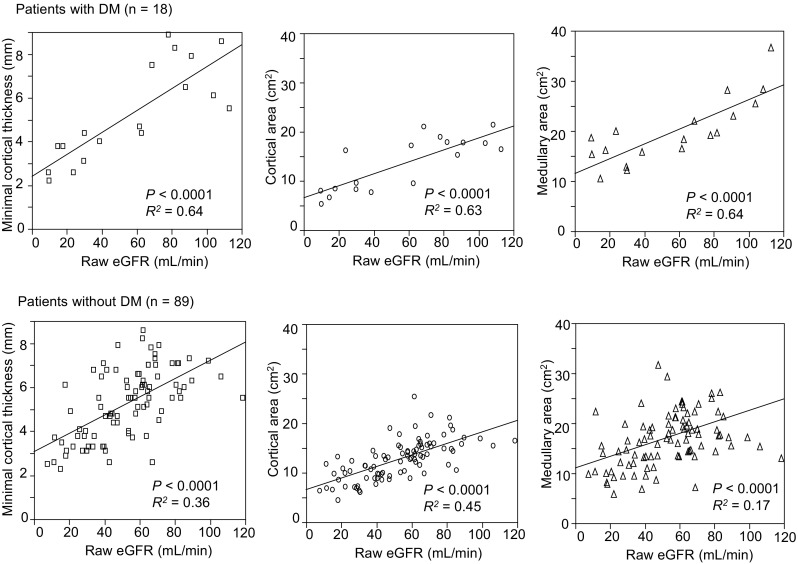
Correlation between raw estimated glomerular filtration rate (eGFR) and minimal cortical thickness, cortical area, or medullary area in patients with chronic kidney disease (CKD) with diabetes mellitus (DM) (*n* = 18) or without DM (*n* = 89). All three kidney morphological parameters showed higher correlation coefficients with raw eGFR in CKD patients with DM than in those without DM

**Fig. 8:**
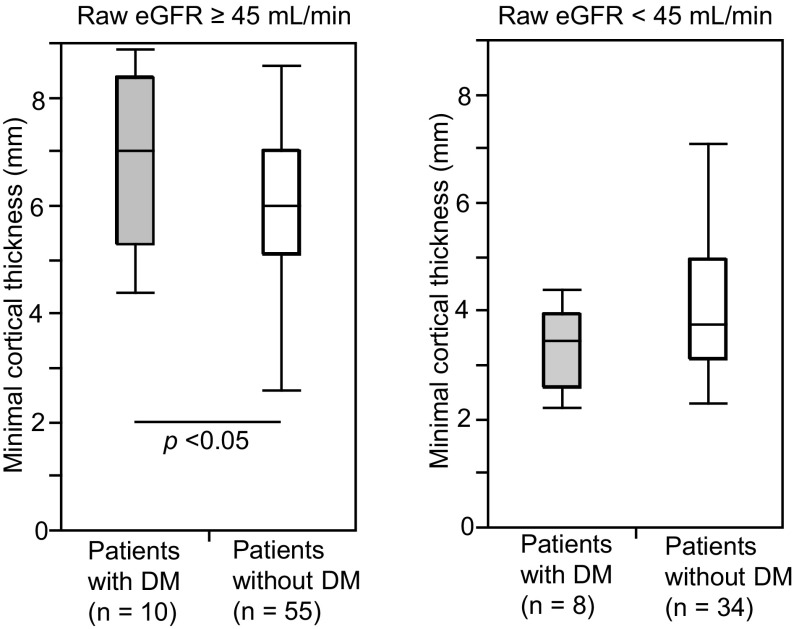
Comparison of minimal cortical thickness between patients with chronic kidney disease (CKD) with or without diabetes mellitus (DM). The patients with CKD were divided into two groups, those with a raw estimated glomerular filtration rate (eGFR) ≥ 45 mL/min and those with a raw eGFR < 45 mL/min, and minimal cortical thicknesses were compared between patients with CKD and DM and those with CKD but without DM. Values are shown as mean ± SD

Finally, we divided the CKD patients into DM (*n* = 18) and non-DM (*n* = 89) groups to evaluate the association between CKD etiology and kidney morphological parameters. Table 4 represents the background profiles of the two groups. Patients with DM were older and had larger body weight, larger body mass index, and larger BSA. HbA1c was also higher in patients with DM. RAS inhibitors were administered to significantly more patients without DM, and larger proportion of patients with DM was treated with diuretics and beta-blockers. Otherwise, no clinical characteristics differed between the two groups. We adopted raw eGFR as a kidney functional marker to evaluate the differences in correlation with morphological parameters between the patients with and without DM since raw eGFR had the highest correlation coefficient with each kidney morphological parameter (Fig. 4). Figure 7 shows that in patients with DM, all three morphological parameters demonstrated higher correlation coefficients with raw eGFR than those in patients without DM. Furthermore, minimal cortical thickness was significantly greater in patients with DM and a raw eGFR ≥ 45 mL/min (Fig. 8), indicating that the atrophic change in the kidney cortex was milder in the earlier stage of CKD in patients with DM.

**Table 4:** Clinical characteristics of the patients with CKD with or without diabetes mellitus

Characteristic	DM (*n* = 18)	Non-DM (*n* = 89)	*P* value
Male sex [*n* (%)]	14 (77.8)	51 (57.3)	0.1047
Age (years; mean ± SD)	67 ± 15	63 ± 18	0.0019
Height (m; mean ± SD)	1.60 ± 0.13	1.60 ± 0.01	0.6772
Weight (kg; mean ± SD)	71.0 ± 20.0	61.3 ± 13.2	0.0113
BMI (mean ± SD)	24.2 ± 4.2	23.7 ± 3.8	0.0026
BSA (m^2^; mean ± SD)	1.91 ± 0.33	1.77 ± 0.23	0.0118
BUN [mg/dL; median (IQR)]	18.1 (13.5–33.1)	17.0 (13.8–21.0)	0.6320
Cre [mg/dL; median (IQR)]	1.08 (0.82–2.42)	1.02 (0.87–1.45)	0.4946
eGFR (mL/min/1.73 m^2^; mean ± SD)	50.0 ± 27.9	51.3 ± 21.6	0.8225
Raw eGFR (mL/min; mean ± SD)	57.2 ± 36.0	53.0 ± 22.9	0.5164
24-h Ccr (mL/min; mean ± SD)	61.5 ± 38.6	68.8 ± 30.2	0.3762
Urinary protein [mg/day; median (IQR)]	458 (136–1610)	126 (48–829)	0.2061
CKD stage [*n* (%)]			0.0699
G1	0 (0.0)	4 (4.5)	
G2	9 (50.0)	26 (29.2)	
G3	4 (22.2)	44 (49.4)	
G4	2 (11.1)	11 (12.4)	
G5	3 (16.7)	4 (4.5)	
HbA1c (%; mean ± SD)	7.1 ± 0.7	5.6 ± 0.3	<0.0001
Anti-hypertensive agents [*n* (%)]	14 (77.8)	79 (88.8)	0.2357
RAS inhibitors [*n* (%)]	8 (44.4)	65 (73.0)	0.0214
Calcium channel blockers [*n* (%)]	10 (55.6)	39 (43.8)	0.3629
Diuretics [*n* (%)]	6 (33.3)	8 (9.0)	0.0120
Beta-blockers [*n* (%)]	8 (44.4)	8 (9.0)	0.0006

*CKD* chronic kidney disease, *BMI* body mass index, *BSA* body surface area, *BUN* blood urea nitrogen, *eGFR* estimated glomerular filtration rate, *Ccr* creatinine clearance, *RAS* renin–angiotensin system

## Discussion

Our study revealed that renal corticomedullary differentiation was more clearly depicted in SSFP MRI with a spatially selective IR pulse than in conventional IP images, even in patients with CKD G4 and G5. This new imaging technique enabled us to measure the thickness or area of the each renal segment without contrast agents in patients with CKD and evaluate renal cortex atrophy.

It has been reported that minimal renal cortical thickness has a positive correlation with renal function because nephrons are mainly located in the renal cortex [16], but the corticomedullary boundary in unenhanced MRI reportedly becomes ambiguous in patients with advanced renal insufficiency, making it difficult to properly measure renal cortical thickness [4, 5]. MRI with the time-spatial labeling inversion pulse method was originally developed to investigate blood flow to ROI by selectively placing the IR pulse and saturating blood spins in the vessels and background [7]. Kanki et al. reported that they applied the SSFP MRI in 20 patients and revealed the improved visibility of renal corticomedullary differentiation by accentuating the differences in T1 values between the renal cortex and medulla without using contrast agents. In SSFP MRI with time-special labeling inversion pulse obtained with optimal TI, protons in the renal cortex with shorter T1 values are substantially magnetized (recovered) while the longitudinal magnetization of protons in the renal medulla with longer T1 values is nulled. Thus, the renal cortex is shown as bright, while the renal medulla is observed as dark, resulting in the better visualization of corticomedullary differentiation [7]. The same research group reported in a series of papers that visualization of the corticomedullary differentiation was significantly better in SSFP images than that on fast asymmetric spin echo images, another method used for noncontrast-enhanced MR angiography [17]. They also reported that this SSFP imaging method was also useful without the influence of aging if optimal TI values for the best visualization were adopted [18]. In the present study, we revealed that corticomedullary contrast ratio was significantly improved by SSFP MRI, even though it was smaller in some cases (Fig. 2). Ideally, optimal TI for each case should be determined for the best visualization of renal corticomedullary junction [7, 18]. In our study, however, we standardized TI for measurement as 1400 ms to compare the corticomedullary contrast ratios under the same condition. Therefore, medulla signals would have been inappropriately higher in some cases.

We also confirmed that morphological parameters demonstrated negative correlation with age (Fig. 6). In a previous study using contrast-enhanced CT, kidney cortical volume alone, but not medullary, was reported to correlate negatively with the age [19]. In the other study, no significant correlation was observed between age and renal cortical thickness measured by SSFP MRI [18]. In these previous studies, patients with decreased kidney function were not included. We surmise that correlation with age and atrophic change of kidney could be confirmed when aged patients with advanced kidney dysfunction were included. Moreover, predicted raw eGFR based on each morphological parameter also significantly correlated with the age, suggesting that SSFP MRI could be substitutable for GFR. We are sure that the present study is complementary to the series of previous reports regarding the effectiveness of SSFP MRI for the more precise evaluation of kidney morphological parameters even in patients with advanced CKD.

On the contrary, the measured morphological parameters had no significant correlations with the urine protein level. This fact may indicate that decreased morphological values reflect chronic atrophic changes but not ongoing glomerular or tubular damage expressed as elevated urine protein level. Morphological changes caused by tubular atrophy and following tubulointerstitial fibrosis would have been valuable indicators for the assessment of residual kidney function and reversibility in progressive chronic kidney disease [20]. We are sure that this morphological assessment would be useful for assessing kidney dysfunction progression.

We also compared the morphological parameters of CKD patients with and without DM and demonstrated that kidney morphological parameters correlated with raw eGFR better in the CKD patients with DM than in those without DM (Fig. 7). We surmised that the kidney atrophic changes of the patients without DM were not homogenous because of the heterogeneity of the etiology of CKD in this group. We also confirmed that minimal cortical thickness was greater in patients with DM with a raw eGFR ≥ 45 mL/min. It has been reported that at the onset of DM, a subset of diabetic patients undergo an increase in GFR caused by glomerular hyperfiltration and kidney growth due to accelerated reabsorption in proximal tubule [21, 22]. Our data were in line with these findings; however, we could not exclude the influence of diuretics, which were administered in significantly larger proportion of the patients with DM. Diuresis could have elevated serum creatinine level, resulting in underestimation of eGFR in patients with preserved kidney size and function.

In the present study, raw eGFR demonstrated the highest correlation coefficient with each of the three kidney functional parameters evaluated in the study. Twenty-four-hour Ccr usually overestimates kidney function due to the tubular secretion of creatinine, especially in patients with advanced CKD and decreased glomerular filtration [23]. The eGFR value is normalized for BSA, but considering that renal cortical volume is positively correlated with BSA and GFR [18, 19, 24], raw eGFR would represent morphological kidney changes in CKD more accurately than eGFR normalized for BSA.

The limitation of this study was that the categorization of patients with CKD with or without DM was not based on the definite diagnosis by kidney biopsy but was clinically different by the presence of DM. The definite histopathological diagnosis by kidney biopsy would be essential to reliably utilize these kidney morphological parameters for the differential diagnosis of the CKD origin using non-invasive SSFP MRI. Another limitation of this study was that this study was performed with 1.5-T unit MRI. Even though 1.5-T unit MRI is mainly used in Japan, 3.0-T unit MRI would be becoming standard. SSFP MRI examination with 3.0-T unit should be conducted in the future study.

In conclusion, noncontrast-enhanced SSFP MRI with a spatially selective IR pulse can improve the visibility of renal corticomedullary differentiation even in patients with advanced CKD patients. This new MRI method could detect early morphological changes in patients with CKD and would be useful for differentiating CKD etiologies in the future.

## References

[1] Cheung CM, Shurrab AE, Buckley DL, Hegarty J, Middleton RJ, Mamtora H, Kalra PA (2006). MR-derived renal morphology and renal function in patients with atherosclerotic renovascular disease. Kidney Int.

[2] Beland MD, Walle NL, Machan JT, Cronan JJ (2010). Renal cortical thickness measured at ultrasound: is it better than renal length as an indicator of renal function in chronic kidney disease?. AJR Am J Roentgenol.

[3] Emamian SA, Nielsen MB, Pedersen JF (1995). Intraobserver and interobserver variations in sonographic measurements of kidney size in adult volunteers: a comparison of linear measurements and volumetric estimates. Acta Radiol.

[4] Semelka RC, Corrigan K, Ascher SM, Brown JJ, Colindres RE (1994). Renal corticomedullary differentiation: observation in patients with differing serum creatinine levels. Radiology.

[5] Lee VS, Kaur M, Bokacheva L, Chen Q, Rusinek H, Thakur R, Moses D, Nazzaro C, Kramer EL (2007). What causes diminished corticomedullary differentiation in renal insufficiency?. J Magn Reson Imaging.

[6] Grobner T, Prischl FC (2007). Gadolinium and nephrogenic systemic fibrosis. Kidney Int.

[7] Kanki A, Ito K, Tamada T, Noda Y, Yamamoto A, Tanimoto D, Sato T, Higaki A (2013). Corticomedullary differentiation of the kidney: evaluation with noncontrast-enhanced steady-state free precession (SSFP) MRI with time-spatial labeling inversion pulse (Time-SLIP). J Magn Reson Imaging.

[8] Noda Y, Ito K, Kanki A, Tamada T, Yamamoto A, Yasokawa K, Higaki A (2015). Measurement of renal cortical thickness using noncontrast-enhanced steady-state free precession MRI with spatially selective inversion recovery pulse: association with renal function. J Magn Reson Imaging.

[9] KDIGO 2012 clinical practice guideline for the evaluation and management of chronic kidney disease. Kidney Int Suppl. 2013;3:19–62. 10.1038/ki.2013.243 23989362

[10] Japanese Society of Nephrology (2014). Evidence-based clinical practice guideline for CKD 2013. Clin Exp Nephrol.

[11] Matsuo S, Imai E, Horio M, Yasuda Y, Tomita K, Nitta K, Yamagata K, Tomino Y, Yokoyama H, Hishida A (2009). Revised equations for estimated GFR from serum creatinine in Japan. Am J Kidney Dis.

[12] DuBois D, DuBois E (1916). A formula to estimate the approximate surface area if height and weight be known. Arch Intern Med..

[13] American Diabetes Association (2010). Diagnosis and classification of diabetes mellitus. Diabetes Care.

[14] Musso CG, Oreopoulos DG (2001). Aging and physiological changes of the kidneys including changes in glomerular filtration rate. Nephron Physiol..

[15] Garasto S, Fusco S, Corica F, Rosignuolo M, Marino A, Montesanto A, De Rango F, Maggio M, Mari V, Corsonello A, Lattanzio F (2014). Estimating glomerular filtration rate in older people. Biomed Res Int.

[16] Mounier-Vehier C, Lions C, Devos P, Jaboureck O, Willoteaux S, Carre A, Beregi J (2002). Cortical thickness: an early morphological marker of atherosclerotic renal disease. Kidney Int.

[17] Kanki A, Ito K, Tamada T, Noda Y, Yamamoto A, Higaki A, Sato T, Yasokawa K, Abe T, Yoshida K (2014). Renal corticomedullary differentiation by non-contrast-enhanced MR imaging with a spatially selective IR pulse at various inversion times: comparison with fast asymmetric spin echo (FASE) and steady-state free-precession (SSFP). Magn Reson Med Sci..

[18] Noda Y, Kanki A, Yamamoto A, Higashi H, Tanimoto D, Sato T, Higaki A, Tamada T, Ito K (2014). Age-related change in renal corticomedullary differentiation: evaluation with noncontrast-enhanced steady-state free precession (SSFP) MRI with spatially selective inversion pulse using variable inversion time. J Magn Reson Imaging.

[19] Wang X, Vrtiska TJ, Avula RT, Walters LR, Chakkera HA, Kremers WK, Lerman LO, Rule AD (2014). Age, kidney function, and risk factors associate differently with cortical and medullary volumes of the kidney. Kidney Int.

[20] Liu Y (2011). Cellular and molecular mechanisms of renal fibrosis. Nat Rev Nephrol..

[21] Vallon V, Thomson SC (2012). Renal function in diabetic disease models: the tubular system in the pathophysiology of the diabetic kidney. Annu Rev Physiol..

[22] Thomson SC, Vallon V, Blantz RC (2004). Kidney function in early diabetes: the tubular hypothesis of glomerular filtration. Am J Physiol Renal Physiol.

[23] Nguyen MT, Maynard SE, Kimmel PL (2009). Misapplications of commonly used kidney equations: renal physiology in practice. Clin J Am Soc Nephrol.

[24] Geddes CC, Woo YM, Brady S (2008). Glomerular filtration rate-what is the rationale and justification of normalizing GFR for body surface area?. Nephrol Dial Transplant.

